# Supply Chain Optimisation for an Ultrasound-Organosolv Lignocellulosic Biorefinery: Impact of Technology Choices

**DOI:** 10.1007/s12649-017-0043-6

**Published:** 2017-08-20

**Authors:** Madeleine J. Bussemaker, Kenneth Day, Geoffrey Drage, Franjo Cecelja

**Affiliations:** 1 Process and Information Systems Engineering Research Centre, Chemical and Process Engineering, University of Surrey, Guildford, GU2 7XH UK; 2 Bio-Sep Limited, Clapton Revel, Wooburn Moor, HP10 0NP UK

**Keywords:** Lignocellulose, Biorefining, Ultrasound, Supply chain optimisation, Techno-economic assessment, Value chain

## Abstract

Conversion of lignocellulose to value-added products is normally focussed on fuel production via ethanol or heat. In this work, a techno-economic assessment of a biorefinery with three product streams, cellulose, hemicellulose and lignin is presented. Moreover, the techno-economic assessment is evaluated in the context of the supply chain through optimisation. A mixed integer linear program was developed to allow for flexible scenarios in order to determine effects of technological and pre-processing variations on the supply chain. The techno-economic and optimisation model integration was demonstrated on a case study in Scotland using woody biomass, either as sawnlogs or sawmill chips. It was established that sawmill chips is the preferred option, however sawnlogs became competitive once passive drying to 30% moisture content (wet basis) was considered. The flexibility of the modelling approach allowed for consideration of technology savings in the context of the supply chain, which can impact development choices.

## Introduction

Biorefinery supply chain analysis, including techno-economic assessment of conversion technologies for lignocellulosic feedstock has traditionally focussed on ethanol or heat production for fuel [1]. Increasingly, however, alternative product streams are being considered, and as biorefining for non-biofuel purposes become more inevitable, tools to evaluate the economic viability of various options are increasingly needed. Previous supply and/or value chain related research efforts have focussed on ethanol production via lignocellulose with specific targets on the distribution of processing hubs [2], comparison of biomass sources [3, 4], first and second generation bioethanol technology assessments [5], weather uncertainties [6] and feedstock properties such as moisture content [7]. In addition, transport cost and distance were demonstrated to be an important consideration for biofuel supply chain networks [8].

Techno-economic analysis of biorefineries has previously considered different ways of utilising the whole lignocellulosic feedstock in order to reduce the minimum ethanol selling price (MESP) and hence to increase the profitability of the process. As demonstrated, ethanol-focussed biorefineries involve a pre-treatment step, aimed at improving the sugar hydrolysis and fermentation yields which normally involves a physical, chemical or biological process, or a combination of these [9]. Pre-treatment for ethanol or fuel production often damages other components in the biomass and as lignin is not used for the ethanol production, it’s value is limited to fuel for electricity to reuse in the ethanol production process [10, 11] or as a local heat source [12]. However, alternate, multi-product biorefinery technology configuration options are now considered to make use of the whole lignocellulosic feedstock for chemicals and value added products, utilising the three core polymers in the feedstock, namely, cellulose, hemicellulose and lignin [13–15]. The multi-product biorefinery system is necessary to capitalise on all of the biomass components, however desires increased purity of the separated core polymers in order to increase the value of these commodities. To this end, a selection of pre-treatment solvents have been considered in techno-economic analyses i.e. ionic liquid treatment [16], organosolv treatments [12, 17, 18] and acid pre-treatment [19] which are used for a variety of products such as ethanol, lignin, furfural, cellulose, succinic acid and acetic acid.

The consideration of co-products in value engineering and target costing of lignocellulosic biorefining is seen as the key to overall economic evaluation and implementation of technology [20]. However, utilisation of the whole biomass presents several key challenges, economically and technologically. The initial pre-treatment step becomes more focussed on fractionation into cellulose, hemicellulose and lignin for future conversion into feedstock chemicals or alternate biobased products [13]. To prevent losses in yields, the pre-treatment step often requires expensive solvent such as organic and ionic liquids to efficiently fractionate the biomass. In order for these solvents to become economically feasible, solvent recycling and lignin valorisation is required [12, 16, 18, 21]. In combination to the pre-treatment solvent, efficient technologies need to be developed to make use of the whole lignocellulose for higher value products [13]. To this end, novel methods for pre-treatment which enhance current treatment options without losses in yields of the three key polymers are required, one of which is ultrasonic processing [22]. Then, to fully appreciate potential cost savings of technologies, or overall feasibility, the cost should be considered in respect to the whole supply chain.

In this work, a flexible, mixed-integer linear model is proposed for optimisation of a biorefining supply chain and for consideration of respective techno-economic assessment. The technology considered is a novel fractionation technique which utilises ultrasound in an organic solvent to aid in the separation of the lignocellulose into lignin, cellulose and hemicellulose. The supply chain model is designed to be flexible to consider: various technology configurations, different operational scenarios with alternate pre-treatments, different biomass properties such as moisture contents, and biomass sources. The model indicates the optimal locations and configuration of the supply chain elements. The optimisation is performed relative to maximising the profit received from the sale of the base product streams, i.e. cellulose, hemicellulose and lignin. Optimisation constraints are set to reflect operational environment and are driven by a set of product demands, feedstock supply implementation scenarios such as fulfilling capacities. The flexibility of the approach is an important novel consideration which reflects the developing scene for the variety of feedstock and product choices in lignocellulosic biorefining. As attention shifts from biofuels to multi-product biorefineries, this flexibility will be required to support decision making for the future biorefining industry.

## Problem Formulation

### Technology Description

**Fig. 1:**
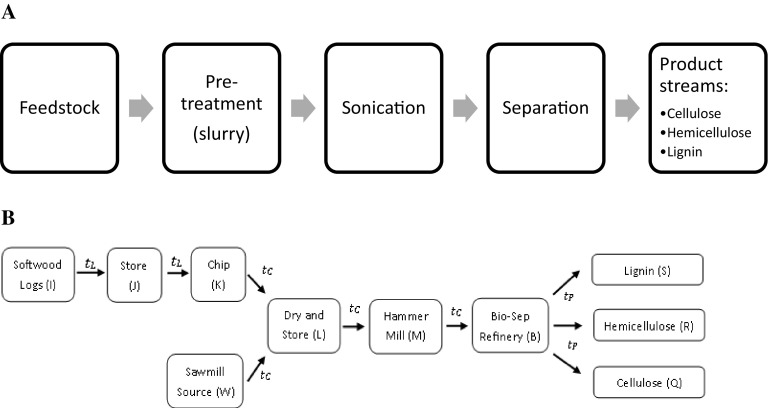
Representation of the two scales modelled. **a** Technology process modelled. **b** Supply chain outline for the Bio-Sep biorefinery

The technology model was developed for a process currently under development by Bio-Sep limited and has been verified at a pilot scale. The heuristic technology model identifies the key bottlenecks of the conceptual design in order to combine these concepts in a holistic supply chain model, described in detail in “Supply Chain Case Study Description” and “Optimisation Model Formulation”. The concept of the two models are presented alongside each other in Fig. 1a, b. The heuristic technology model was developed in Microsoft Excel, using criteria from a verified experimental design. The heuristic technology model was developed using mass and energy balances, and accounts for prices and processing parameters obtained experimentally. Due to commercial sensitivity, only limited set of data is presented in this paper. The key elements of the technology include a feedstock input, pre-treatment to make a slurry, sonication and separation into three product streams: cellulose hemicellulose and lignin, as represented in Fig. 1a.

#### Pre-treatment: Slurry Preparation

At the pilot scale, the slurry preparation, the pre-treatment stage, involves mixing biomass at a rate of 0.25 ton per hour (5 ton/day), as received in the correct size increments of 3–5 mm, with a dilute organic acid solution. The feedstock moisture content used is in the range of 10% (wet basis) and the addition of aqueous solution achieves a solid to liquid ratio of about 1:10 (w:w). The mixture is then heated (80 °C) prior to blending and de-aeration, after which ethanol and methyl isobutyl ketone (MIBK) are added to produce the organosolv mixture (acidic water:ethanol:MIBK, 50:34:16). During slurry preparation, the mixture is continuously circulated and then moved to the sonication stage. The energy required to heat the slurry and the resultant temperature after the addition of MIBK and ethanol are calculated from specific heat capacities and weights of each component of the mixture. Heat loss is allowed for: 10% during blending/de-aeration and 20% at the pumping steps.

#### Sonication

The sonication stage involves two sonication and separation steps, the second with fresh organosolv solution. Once the slurry is pumped into the ultrasonic reactor, the mixture is further heated prior to ultrasonic treatment (120 °C). After sonication the mixture is cooled (40 °C) to prepare for solid liquid separation by centrifuge. During heating, sonication and cooling the mixture is continuously circulated. After solid liquid separation, the liquid is kept for later extraction and purification, while the solid is passed through sonication again. The organosolv mixture is added to the solid in a solid to liquid ratio of about 1:20 (w:w) prior to heating to reaction temperature once more. The solution is again sonicated and cooled with continuous circulation. Possible heat gain during sonication based on ultrasonic heat input is taken into account. The solid to liquid ratio is designed to be consistent with the solid to liquid ratio present during the first sonication stage. After the second centrifuge the solid is ready for subsequent processing and the liquid is combined with the liquor produced after the first solid liquid separation.

#### Separation

Separation refers to the treatment of the liquid and solid streams after centrifuging. The solid stream is dried and the residual liquid remaining in the solid stream is counted as lost. The organic and aqueous fractions are separated using a predefined formula [23] which involves the addition of water to induce a phase separation. The organic fraction contains the lignin and MIBK from which MIBK is removed by membrane or distillation technology in a short cycle (under an hour). The aqueous fraction contains the acidic water, ethanol and hemicellulosic sugars. The ethanol and water are removed and separated by membrane or distillation technology again in a short cycle prior to sugar separation using chromatography. The recovery of cellulose, lignin and hemicellulose is assumed, based on preliminary experiments, to be 98, 72 and 94, respectively. The recovery of the solvents, MIBK and ethanol can be varied to investigate economic impact and/or to reflect the particular operational condition.

The separated water then contains impurities and organic acid. The addition of water for phase separation decreases the organic acid concentration compared to the required charge for the initial acid treatment, hence recycling is not economically feasible. The acid is neutralised with sodium carbonate and the cost accounted for in the supply chain. The neutralisation reaction was used to calculate the carbon dioxide and salt product. The CO_2_ mass is comparatively small but may need to be taken into account in lieu of CO_2_ tariffs, which have been set at a £10/ton (2012, GBP).

#### Economic Analysis

The techno-economic model was used to assess the operational performance and with the focus on the key costs in the technology. The operational costs are evaluated in order to highlight the most economical configuration of the technology. Details on the operational costs are provided in the “Appendix”. Then, this information is used to evaluate the technology within the biorefinery supply chain with the additional consideration of capital costs and product revenue. The operational cost takes into account the costs saved by product recycling as well as the heat, electricity and materials.

### Supply Chain Case Study Description

**Fig. 2:**
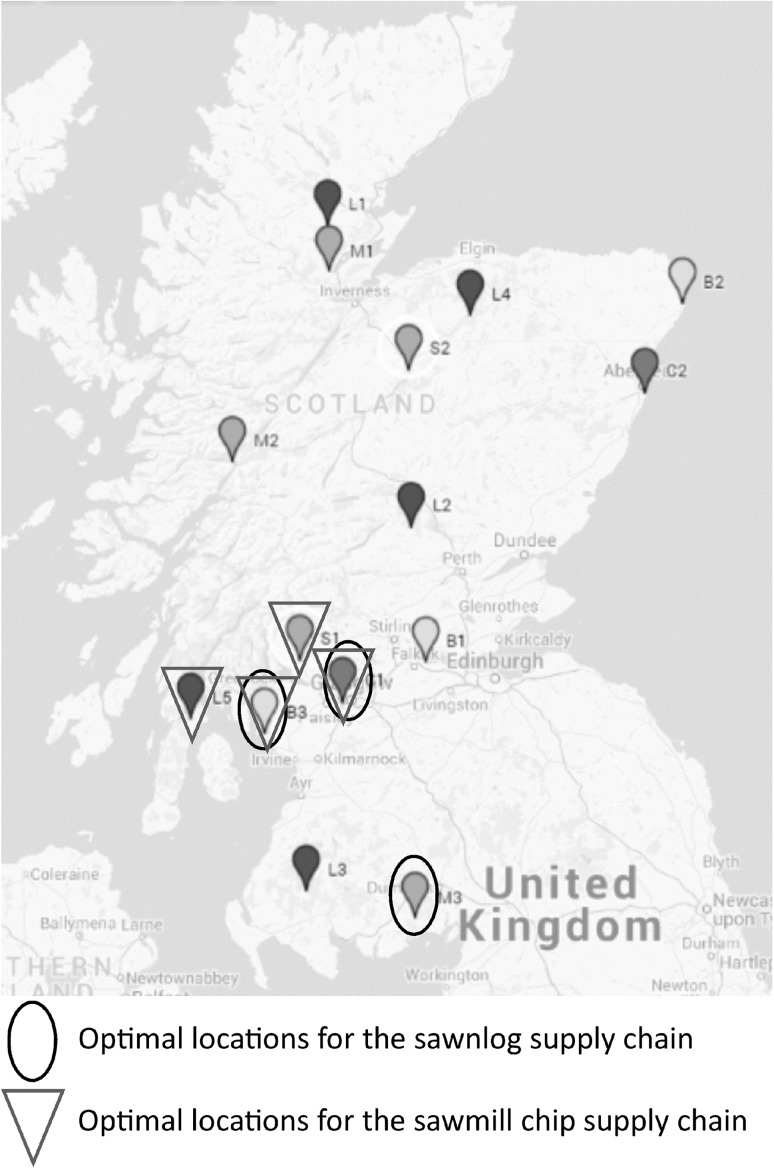
Candidate points for the base case scenario with two optimal solutions, using sawmill chips versus sawnlogs. Log sources, L1-5; log stores, S1-2, sawmills, M1-3; biorefineries, B1-3; and customers, C1-2

The model was developed using a case set in Scotland with the use of softwood either as logs or as chips from the sawmill by-product. The prices in the model are based on the pound sterling scaled to 2012. The candidate points are shown in the map in Fig. 2, alongside the respective optimal solutions and the outline of the capital and operational costs modelled are shown in Table 1.

**Table 1:** Outline of capital and operational costs modelled

Supply chain node	Capital costs	Operational costs
Store (J)	Land rental	Caretaker fees per tonne
Chip (K)	Purchase of chipper Insurance Personnel	Repair and maintenance Energy
Dry and store (L)	Land Fixtures Personnel	Energy
Hammer mill (M)	Land Fixtures Personnel Mill	Energy Repair and maintenance

#### Candidate Point Selection and Transportation

The pre-processing operations, chipping, drying and milling could either be located at the log storage sites, the sawmill sources or the biorefinery locations. The biorefineries were co-located with existing power plants to explore the potential benefit of using the excess heat from the power plant in the context of the supply chain. Log storage sites were selected based on feasible land areas, located next to main roads to serve the log sources in the forest. Existing sawmill locations were used for chip sources and for sawn log sources, areas central to forest regions were chosen as a representation of the forest log collection points. The data for each route type was made on collation of information collected for the Road Haulage Association [24]. Diesel fuel cost was averaged over 2012, as reported by the UK government, (1.4216 £/L) with a summary of costs and capacities given in Table 2. Changes in moisture content, effecting transportation costs are accounted for using the difference in biomass weight. Distances were calculated using the Haversine formula as per Eq. (1) which correlates well with comparative mapped road distances.

**Table 2:** Transport pricing calculated according to UK data [24]

Transport type	Distance (£/ton/km)	Load (£/ton)	Capacity (km ton/year)
Forest logs	0.53	6.57	2.42 × 10^6^
Highway logs	0.25	6.57	2.42 × 10^6^
Chips and milled feed stock	0.15	1.88	2.51 × 10^6^
Products—large trucks	0.31	4.37	4.34 × 10^5^
Products—small trucks	0.46	6.59	2.53 × 10^5^

1 $${\text{haversin}}\left( {\frac{d}{r}} \right) = {\text{haversin}}~\left( {\emptyset _{a} - \emptyset _{b} } \right) + {\text{cos}}\left( {\emptyset _{a} } \right){\text{cos}}\left( {\emptyset _{b} } \right){\times\text{haversin}}\left( {\lambda _{b} - \lambda _{a} } \right)$$

where

2 $${\text{haversin}}\left( {\theta} \right)={\text{si}}{{\text{n}}^2}\left( {\frac{\theta}{2}} \right)$$

*d* = the distance between two points, *a* and *b, r* = the earth’s radius, ${\emptyset _a},{\emptyset _b}$ latitudes of point *a* and *b*, ${\lambda _a},{\lambda _b}$ longitude of point *a* and *b*.

#### Feedstock Sources (F)

Trees are bought as sawnlogs at an average price of £47.60 per green ton [25] with a moisture content (MC) of 60% (wet basis) for Sitka Spruce [26]. However, if any tree is allowed to air dry, the moisture content can be reduced to 30% in the period of 18 months [27]. Here the price per cubic metre is converted using the mass ratios to a related price of £92/ton (30% MC), with a nominal 10% added to incorporate cost of passive drying.

The sawmill availability is based on three different sawmills in Scotland, considering their annual production of by-products. The sawmills were assumed to provide green chips with 55% MC (wet basis) [27]. The wet wood price taken for woodchips was £30 which was an average of public and private sources provided which was corrected for any dryer prices using a mass conversion and adding 10% nominal additional holding cost per ton.

#### Pre-processing Stages (O)

The figures for capacity, fixed cost and operational cost of the various pre-processing costs are summarized in Table 3. Estimates were made for the cost of a storage facility (J) with capacity of 1200 tons at one time. This was estimated to require 0.5 acres and land values were used to estimate the cost of the land use per year. The operational cost was estimated based on the requirement of a caretaker for 8 h per fortnight on minimum wage.

**Table 3:** Fixed costs and capacities of different pre-processing stages

Pre-process	Capacity (ton/year)	Fixed cost (£/y)	Operational cost (£/ton)
Storage (J)	20,000	200	1
Chipping (K)			
At log store	23,500	48,300	0.95
At BioSep facility	44,000	64,000	0.95
Milling (M)			
At dry/store	5040	20,100	3
At Bio-Sep site	18,000	31,800	3

Each downsizing (chipping and milling) stage had two capacities, one at the Bio-Sep facility which was based on working similar hours to the Bio-Sep facility (20 h per day, 44 weeks per year, 7 days per week) or at another location with normal working hours (47 weeks per year, 5 days a week, 7 h a day).

The chipping scenario costs were based on chipping costs with respect to forest logs. The chippers were assumed to have a lifetime of 5 years, and estimates of personnel (£15/h) and insurance (£1/h) included in the analysis. Operational cost included repair and maintenance allowance (£10/h) and fuel cost, (£12.48/h) dependent on the capacity [28]. These were converted from pounds per hour to pounds per ton of input material.

Milling capacities were considered (Zhangqiu Yulong Co. Ltd), with an assumed conversion rate of 95%. The milling lifetime was assumed to be 10 years, and capital cost included maintenance and depreciation, (20% of capital) electricity requirements (£3/ton) and labour. Labour was 50% of a full time labourer, at either the sawmill or the Bio-Sep facility. It was assumed that the labourer also contributed to maintenance costs of the mill.

#### Chip Dry and Store (L)

The user must ensure that there are appropriate drying facilities for the given moisture content of the feedstock, ${M}_{oc}$. Then the weight conversion, ${C}_{l}\left(L\right)$ of the drying facilities is calculated using a rearrangement of the moisture content calculation, on a wet basis, as used by the forestry commission (Eq. 3) [29]. The calculation calculates the conversion rate for the initial mass, ${m}_{1}$, with moisture content ${M}_{oc1}$ to the new mass, ${m}_{2}$ with desired moisture content, ${M}_{oc2}$ (Eqs. 4, 5).

3 $$M_{{oc}} = \frac{{{\text{mass}}\,({{\text{wet}}}) - {\text{mass}}\,({{\text{dry}}})}}{{{\text{mass}}\,({{\text{wet}}})}}$$

4 $${C_l}\left( L \right)=\frac{{100 - {M_{oc1}}}}{{100 - {M_{oc2}}}}$$

5 $${m_2}={C_l}(L) \times {m_1}$$

Woodchip drying can normally be facilitated to 10% MC based on woodfuel drying, and pelletisation reports [27, 29, 30]. Average energy use for drying woodfuel pellets were 11 kWh per % of MC reduction to 10% MC. Then, converting to relevant costs used in this study, the calculated cost per dry ton equivalent (dteq) per percent reduction in moisture content was c.a. £1.00/dteq/% MC reduction. This value was translated into a cost per input (wet ton), to reach 10% MC as displayed in Table 4. Here, a degree of linearity in the drying rate is assumed which is imperfect and hence moisture content is investigated through the optimisation model. Then, the cost of larger scale dryers was estimated, with an additional land and labour cost, which gave a total of £22,600/year [31]. The capacity of each drying site was then based on an estimated drying capacity of a total tonnage of moisture removal of 4.5 kilotons of moisture per year.

**Table 4:** Drying costs

Moisture content (MC) (%)	Operating cost [£/ton(wet)]	Capacity (wet ton/year)	Conversion rate
60	125	9000	0.44
55	100	10,000	0.50
50	80	11,250	0.56
40	50	15,000	0.67
30	29	22,500	0.78
10	0	400,000 (nominal)	1

Variations per moisture content to reduce to 10% moisture

#### Product Values

There are three main products for consideration in the supply chain, namely; lignin, cellulose and hemicellulose. Mid-range values for cellulose, lignin and hemicellulose were used at £2600, £460 and £1200, per ton respectively. This was based on the organosolv lignin valorisation, [32] cellulose values from the National Non-Food Crops Centre (NNFCC), [33] and online [31] and an approximation for hemicellulose using, xylose values [31].

### Optimisation Model Formulation

The supply chain model is designed to have two possible input routes, raw agricultural or forestry residues and/or industrial residues. This configuration was initially developed from setting the scene of biorefining in an area of timber production where the biorefinery may either utilise raw wood or sawmill by-products as feedstock. However, the model can also be adapted to agricultural scenarios. In this case, forestry sources were focussed on using the softwood log source and chipped feedstock source from the sawmill (Fig. 1b). For the sawnlog source, the logs were stored, then chipped prior to a drying and storage stage. The sawmill source was assumed to be chipped wood and hence was transported directly to the drying and storage stage. After drying, milling or alternate pre-processing is allowed for before refining into three product streams, lignin, hemicellulose and cellulose. The relevant parameters used in the supply chain model are detailed in Table 5.

**Table 5:** Parameters and variables used in the optimisation model

Decision variables			
Material transported via transportation route between nodes, *σ* $\in$ * ϕ* and *ψ* $\in$ $\varPsi$			${x}_{\sigma ,\psi }\in {X}_{\varPhi ,\varPsi }$
Binary decision variables			
Existence of pre-processing	${E}_{O}\left(O\right)$		
Existence of biorefinery at N	${E}_{B}\left(N,B\right)$		
Supply chain and transportation parameter sets			
Log source points	$i\in I$	Transportation type	$t\in T$
Sawmill source points	$w\in W$	Distances between stages	${{d}_{\sigma ,\psi }\in D}_{\varPhi ,\varPsi }$
Feedstock sources	$\left\{I,W\right\}\in F$	Biorefining locations	$n\in N$
Storage locations	$j\in J$	Set of biorefineries	$b\in B$
Chipping locations	$k\in K$	Cellulose buyer	$q\in Q$
Dry and store locations	$l\in L$	Hemicellulose buyer	$r\in R$
Milling locations	$m\in M$	Lignin buyer	$s\in S$
Pre-processing stage	$\left\{J,K,L,M\right\}\in O$	Product buyer	$\left\{Q,R,S\right\}\in Z$
Conversion parameters			
Pre-processing conversion rate	${C}_{O}\left(O\right)$	Conversion to hemicellulose	${C}_{R}\left(B\right)$
Conversion to cellulose	${C}_{Q}\left(B\right)$	Conversion to lignin	${C}_{S}\left(B\right)$
Cost and income parameters			
Cost of feedstock	${P}_{p}\left(F\right)$	Operational pre-processing cost	${P}_{po}\left(O\right)$
Transport load cost	$Pl\left(T\right)$	Fixed cost of biorefinery	${P}_{bf}\left(B\right)$
Transport distance cost	$Pt\left(T\right)$	Operational biorefinery cost	${P}_{bo}\left(B\right)$
Fixed cost of pre-processing	${P}_{pf}\left(O\right)$	Price of product	${P}_{z}\left(Z\right)$
General capacity parameters			
Availability of feedstock	${A}_{F}\left(F\right)$	Capacity of the biorefinery	${A}_{N}\left(B\right)$
Capacity of transport	${T}_{c}\left(T\right)$	Capacity of buyer	${A}_{z}\left(Z\right)$
Capacity of pre-processing	${A}_{p}\left(O\right)$		
Exclusive capacity parameters			
Minimum biorefinery capacity	${AM}_{N}\left(B\right)$	(Technology-limited)	
Minimum feedstock supply	${AM}_{F}\left(F\right)$	(Supply-driven)	
Minimum buyer demand	${AM}_{z}\left(Z\right)$	(Demand-driven)	

The objective of the model was to maximise profit and this was achieved by applying the mixed integer linear programming (MILP) optimisation of the general form:

6 $$\begin{aligned} {\text{maximise}}\quad & {\text{Profit}} = f{\text{(}}X{\text{)}} \\ {\text{s.t.}}\quad & h{\text{(}}X{\text{)}} = {\text{0}} \\ & g{\text{(}}X{\text{)}} \le {\text{0}} \\ & E_{x} \in \left\{ {0,1} \right\} \\ \end{aligned}$$

where the objective function *f* as well as the constraints *g* and *h*, are all functions of ${X}_{\varPhi ,\varPsi }$ which refers to the material flow between two nodes, *ϕ* and $\varPsi$ in the supply chain. The set of binary variables ${E}_{x}$ defines existence of processing operations ($x=O$) and biorefinery operations ($x=B$). The model was developed in Micosoft Excel using the Solver Foundation with the Gurobi optimiser.

The objective function Eq. (7) is calculated from the difference between costs and income:

7 $${\text{Maximise}}\sum {\text{Income}} - \sum {\text{Cost}}$$

The costs are calculated from purchasing of raw material, Eq. (8) costs of each processing stage of biomass (Eqs. 12, 13, 17, 18) and transport costs, (Eqs. 24, 25). Then the income is calculated from the sales of products to the market (Eq. 22).

#### Feedstock Provision

The feedstock purchased is either softwood logs or sawmill by-products. The feedstock purchase cost [${P}_{p}\left(F\right)$] is dependent on the two different sources, log sources (I) or sawmill sources (W), collectively denoted feedstock sources (F). Hence total feedstock cost is detailed in Eq. (8):

8 $$\sum {P}_{p}\left(F\right)\cdot{X}_{F,\varPsi }$$

And the cost of feedstock for each source can be separated into sawmill source (Eq. 9) and log source (Eq. 10):

9 $$\sum {P}_{p}\left(W\right)\cdot {X}_{W,{L}}$$

10 $$\sum {P}_{p}\left(I\right)\cdot {X}_{I,{J}}$$

The amount of feedstock used is limited by the availability of each feedstock at each location [${A}_{F}\left(F\right)$] giving the feedstock availability constraint:

11 $$\sum {X_{{F,\varPsi }} } \le A_{F} \left( F \right),\quad \forall i,w \in \left\{ {I,W} \right\} \in F$$

#### Pre-processing Stages

Then, each pre-processing stage outlined in Fig. 1b consists of operational and capital costs, as outlined in Table 1 for storage, chipping, drying and milling, with the refining costs outlined previously. The operational costs $({P}_{po})$ for each pre-processing stage (*O*) are dependent on the amount of material sent to the pre-processing stage:

12 $$\sum {P}_{po}\left(O\right)\cdot {X}_{\varPhi ,O}$$

And the fixed costs [${P}_{pf}\left(O\right)$] are based on the estimated capital expenditure, using the binary decision variable on existence of the particular pre-processing stage:

13 $$\sum {P}_{pf}\left(O\right)\cdot {E}_{O}\left(O\right)$$

Each pre-processing stage has associated capacity [${A}_{p}\left(O\right)$] which cannot be exceeded, giving the capacity constraint, using the existence variable:

14 $$\sum {X}_{\varPhi ,O}\le {A}_{p}\left(O\right)\cdot {E}_{O}\left(O\right),\quad\forall j,k,l,m\in \left\{J,K,L,M\right\}\in O$$

In addition, there can only be one type of each pre-process at each location:

15 $$\sum {E}_{O}\left(O\right)\le 1,\quad\forall j,k,l,m\in \left\{J,K,L,M\right\}\in O$$

Note that locations are defined for each stage of the pre-processing ($j,k,l,m\in \left\{J,K,L,M\right\}$). Hence the set of locations for *K* may include locations at the same site as *J*. Therefore consecutive processes may occur at the same location but two of the same processes cannot. Then, the material flow away from each node cannot exceed what is transported to the node, in consideration of the conversion rate [${C}_{o}\left(O\right)$]:

16 $$\sum {X}_{O,\varPsi }\le \sum {X}_{\varPhi ,O}\cdot {C}_{o}\left({O}\right),\quad\forall j,k,l,m\in \left\{J,K,L,M\right\}\in O$$

#### Biorefinery Stage

The biorefinery stage is modelled following similar methodology to the pre-processing stages, whilst allowing for some additional flexibility. The biorefinery stage is subject to operational cost (${P}_{bo}$), dependent on how much biomass is sent to the biorefinery (${X}_{M,N}$):

17 $$\sum {P}_{bo}\cdot {X}_{M,N}$$

Then, the fixed cost (${P}_{bf}$) are accounted for using the existence variable (${E}_{B}$):

18 $$\sum {P}_{bf}\cdot {E}_{B}$$

In this case, the model was also able to choose the biorefinery type (*B*) to be located at the biorefinery candidate point locations (*N*). Only one biorefinery could be located at each candidate point:

19 $$\sum {E_{B} } \left( {N,B} \right) \le 1,\quad\forall b,n \in B,N$$

This allowed for the comparison of the centralised, single large facility compared to the distributed smaller facilities. Then, the capacity of the biorefinery had to be considered, giving the capacity constraint:

20 $$\sum {X}_{M,N}\le {A}_{N}\left(B\right)\cdot {E}_{B}\quad\forall b,n\in B,N$$

Then the material flow balance was included, using different conversion rates [${C}_{Q}\left(B\right),{C}_{R}\left(B\right),{C}_{S}\left(B\right)$] for each product stream ($q,r,s\in \left\{Q,R,S\right\}\in Z$), such that what is transported away from biorefinery must be less than or equal to what was produced:

21 $$\sum {X}_{N,Z}\le \sum {X}_{M,N}\cdot {C}_{Q}\left(B\right)+\sum {X}_{M,N}\cdot {C}_{R}\left(B\right)+\sum {X}_{M,N}\cdot {C}_{S}\left(B\right),\quad\forall m,n,z\in M,{N},Z$$

#### Product Nodes

Income includes received value from the products sold, lignin, hemicellulose and cellulose:

22 $$\sum {P}_{z}\cdot {X}_{N,Z}$$

where each product, in this case, cellulose, hemicellulose and lignin are denoted *Q, R, S*, respectively and are subsets of the total product set, *Z*. Each buyer has a capacity [${A}_{z}\left(Z\right)$] which cannot be exceeded giving the buyer capacity constraint:

23 $$\sum {X}_{N,Z}\le {A}_{z}\left(Z\right)\quad\forall q,r,s\in \left\{Q,R,S\right\}\in Z$$

#### Transportation

Transport costs are accounted for with respect to load carried over the relevant distance. This was dependent on the distance travelled (${D}_{\varPhi ,\varPsi }$), weight transported (${X}_{\varPhi ,\varPsi }$) and cost of transportation per weight and distance [$Pt\left(T\right)$]:

24 $$\sum Pt\left(T\right)\cdot {X}_{\varPhi ,\varPsi }\cdot {D}_{\varPhi ,\varPsi }$$

where the set* T* denotes the transportation available, restricted to different stages of the supply chain. For instance log transportation, chip transportation and product transportation. Similarly, each transportation type has associated loading cost [$Pl\left(T\right)$] dependent on the weight of the load (${X}_{\varPhi ,\varPsi }$) resulting in the overall cost:

25 $$\sum Pl\left(T\right)\cdot {X}_{\varPhi ,\varPsi }$$

The capacity of the transportation type is also defined (${T}_{c}$) for each transportation type and cannot be exceeded:

26 $$\sum {X}_{\varPhi ,\varPsi }\le {T}_{c},\quad\forall t\in T$$

#### Optimisation Constraints

The model optimises the selection of geographical locations of storages, pre-processing sites and biorefining site(s), selection of customers, as well as the transportation routes and modes according to the minimum requirement dictated by the chosen scenario from three different considered; technology-limited, demand-driven and supply-driven scenarios, Eqs. (27–29). The technology-limited scenario assesses the supply chain given a certain technology and includes a constraint that the minimum capacity of the technology [${AM}_{N}\left(B\right)$] has to be met:

27 $$\sum {X}_{M,N}\ge {AM}_{N}\left(B\right),\quad\forall b,n\in B,N$$

The demand-driven scenario dictates that a certain demand for one, two or all products [${AM}_{z}\left(Z\right)$] must be met within the capacity of each stage of the supply chain:

28 $$\sum {X}_{N,Z}\ge {AM}_{z}\left(Z\right),\quad\forall q,r,s\in \left\{Q,R,S\right\}\in Z$$

Lastly, the supply-driven scenario dictates that a certain amount of raw material sawnlogs and/or sawmill by-products [${AM}_{F}\left(F\right)$] must be used:

29 $$\sum {X}_{F,\varPsi }\ge {AM}_{F}\left(F\right),\quad\forall i,w\in \left\{I,W\right\}\in F$$

Each scenario was considered exclusively for different goals of the analysis. Hence constraints (Eqs. 27–29) were applied one at a time to obtain various optimisation results.

## Results and Discussion

### Techno-Economic Analysis

**Fig. 3:**
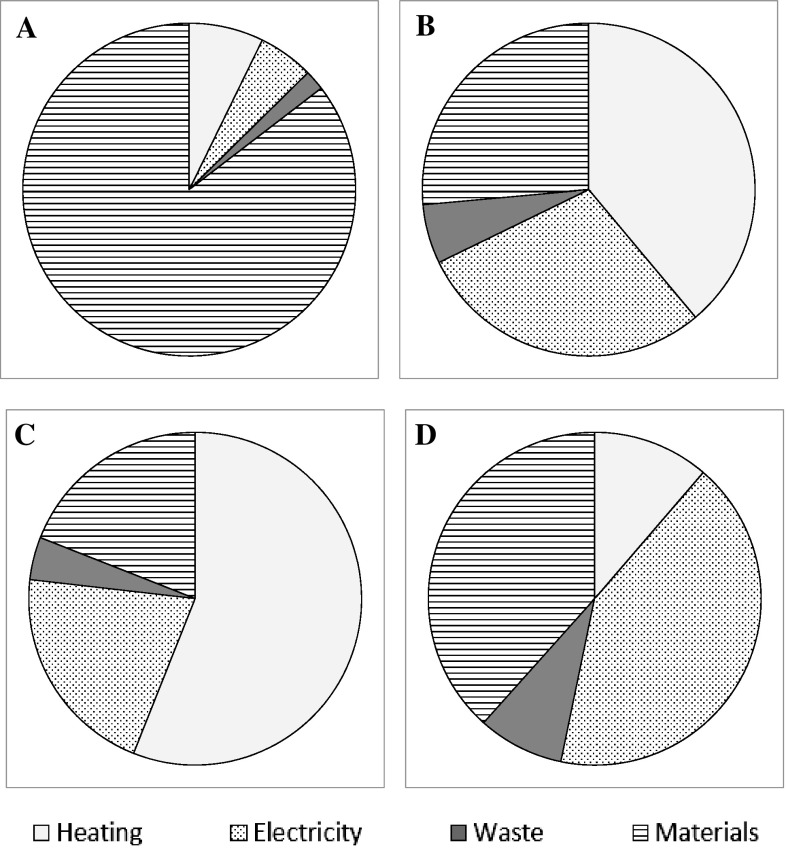
Operational cost distributions in various scenarios. **a** 76% EtOH recovery, 93% MIBK recovery, electricity price £0.10/kWh and heating cost of £0.05/kWh. **b** 99% EtOH recovery, 99% MIBK recovery, electricity price £0.10/kWh and heating cost of £0.05/kWh. **c** 99% EtOH recovery, 99% MIBK recovery, electricity price £0.10/kWh and heating cost of £0.10/kWh. **d** 99% EtOH recovery, 99% MIBK recovery, electricity price £0.10/kWh and heating cost of £0.01/kWh

**Fig. 4:**
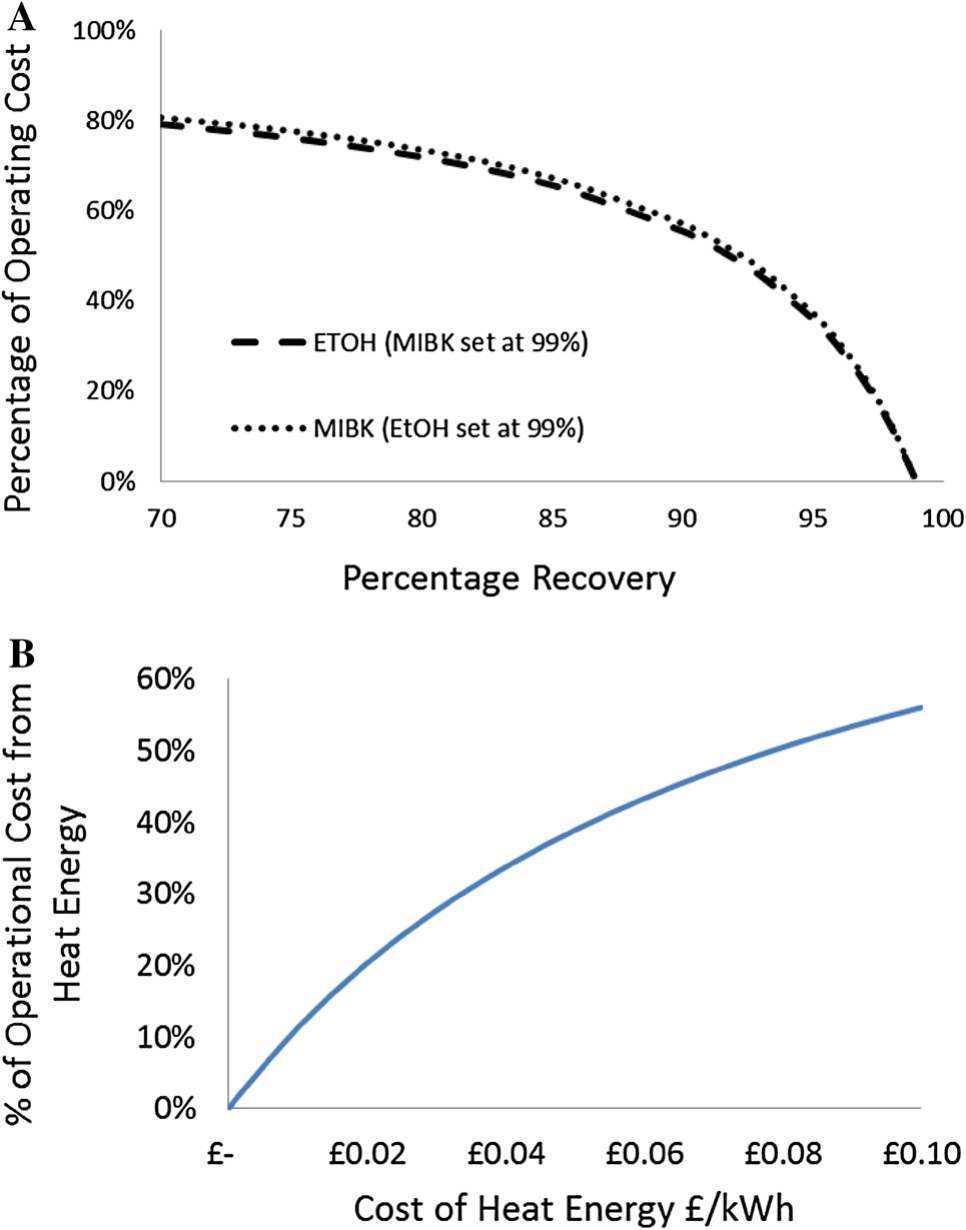
Results from the techno-economic analysis of the Bio-Sep Process. **a** The percentage of operational cost of ethanol (ETOH) and MIBK. The percentage recovery was varied from 70 to 99% and in each case the recovery of the other solvent was set at 99%. **b** Contribution of heat energy to total operational cost at different energy prices. The solvent recovery percentages were set at 99% for both EtOH and MIBK for this analysis

The techno-economic analysis was developed based on assumption of 76% recovery of MIBK and a 93% recovery of ethanol following experimental results and literature values [21]. This gave a proportional operational cost contribution with 85% of the total cost being the cost of organic solvents (Fig. 3a) and a total operating cost of £1913/tone. However, typical solvent recoveries can vary, for example ethanol recovery has been cited at 75% [17] and 93% [21] in an ethanol water ultrafiltration refinery. Hence contribution of the material cost of solvent with varying recovery, relative to the operational cost is also modelled to further explore the key economic bottleneck of the technology (Fig. 4a). Assuming a 99% recovery of MIBK, the cost of ethanol is 79% of the total operating cost with 70% recovery. Similarly, if we assume a 99% recovery of ethanol, MIBK contributes to about 81% of the operational cost with 70% recovery. In both cases, the contribution to cost rapidly decreases when recovery is increased between 90 and 100% compared to the slow decrease between 70 and 90% recovery (Fig. 4a). The importance of solvent recovery in biorefining economics is also confirmed by previous assessment of processes utilising either an organic solvent or more expensive solvents such as ionic liquids [12, 16, 17]. However, it should also be noted that the lignin recovered using these solvents has increased purity and the likely economic value would also be increased [32]. The increased lignin value is not always taken into consideration of the process economics but may serve to offset recovery requirements for a positive economic outcome. The continued development of the technology is directed towards a higher solvent recovery of 99%. This reduces the contribution of solvents to the total cost, dependent on the heat price used (Fig. 3b–d).

A high solvent recovery (99%), demonstrates that the heating cost is the next highest contributor to the total operational cost of 30% (Fig. 3c) with a total cost of £356/ton. In the initial scenario, electricity is assumed to have operational cost of £0.1/kWh and gas sourced heating at £0.05/kWh. In reality the heating price may vary with the location of the plant depending on the availability of heat sources. Hence, the effect of costing the heating from £0.01 to £0.1/kWh was theoretically explored with assumed solvent recoveries of 99% for MIBK and ethanol. The contribution to the operational cost is shown in Fig. 4b. With a reduction in price from £0.10 to £0.01/kWh the total operational cost becomes £254/tonne, compared to £485 and £356/tonne for heat priced at £0.10 and £0.05/kWh, respectively. The contribution of heat energy to total operational cost is reduced with heat price: 56, 39% then to 11% for heat price of £0.10, £0.05 and £0.01/kWh, respectively. The effect on cost distribution is shown in Fig. 3b–d. The figure demonstrates that, once these savings are taken into consideration operational cost is then largely contributed to by materials and electricity. Of the electrical cost, 63% comes from the ultrasonic energy cost.

Ultrasound is a relatively new technology and its use for lignocellulosic conversion has increased in recent years. However, as ultrasound is still in the nascent stage, there is room for more efficient utilisation of the ultrasonic energy through parametric variation such as gas environment, frequency settings, reactor design and configuration [34, 35]. It has been shown that reactors can be designed for optimum power dissipation, flow configuration, wave attenuation and mixing time, all of which contribute to the ultrasonic effects and required power usage [36]. Consequently, a high contribution of energy usage from ultrasound is expected to decrease and will be targeted in future configurations of the technology.

**Fig. 5:**
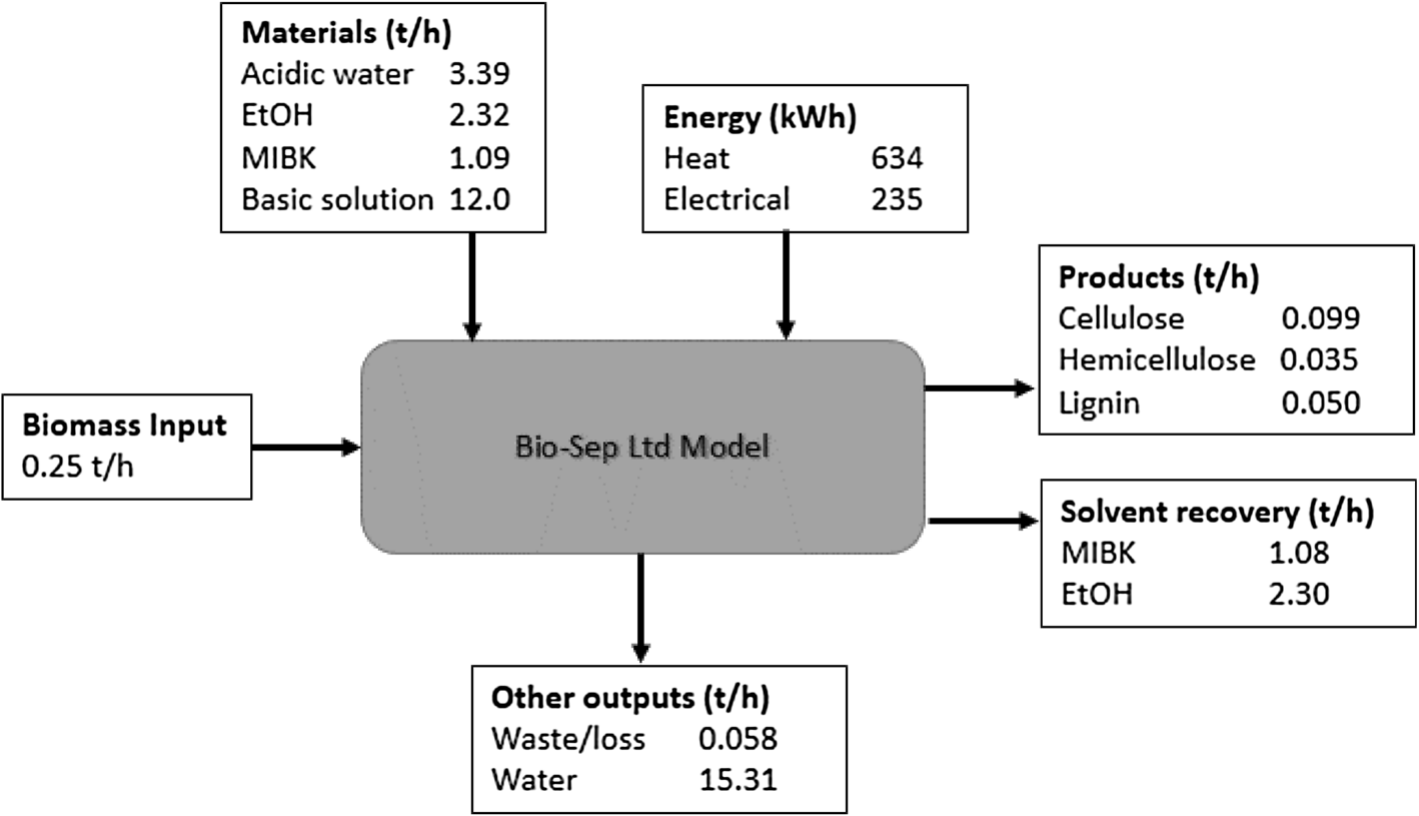
Key inputs and outputs from the techno-economic model of the Bio-Sep Ltd technology

The base case considered for the technology for the optimisation scenario is to assume the solvents are recycled with 99% efficiency. A summary of the key inputs and outputs is presented in Fig. 5. As a starting point the heating cost was assumed to be £0.50/kWh. This assures a total fixed cost (in both cases) of £753,000 and an operational cost of £485, £358 and £257 year/tonne for heating at £0.10, £0.05 and £0.01 kWh, respectively.

### Supply Chain Optimisation Results

#### Variations in Moisture Content

**Fig. 6:**
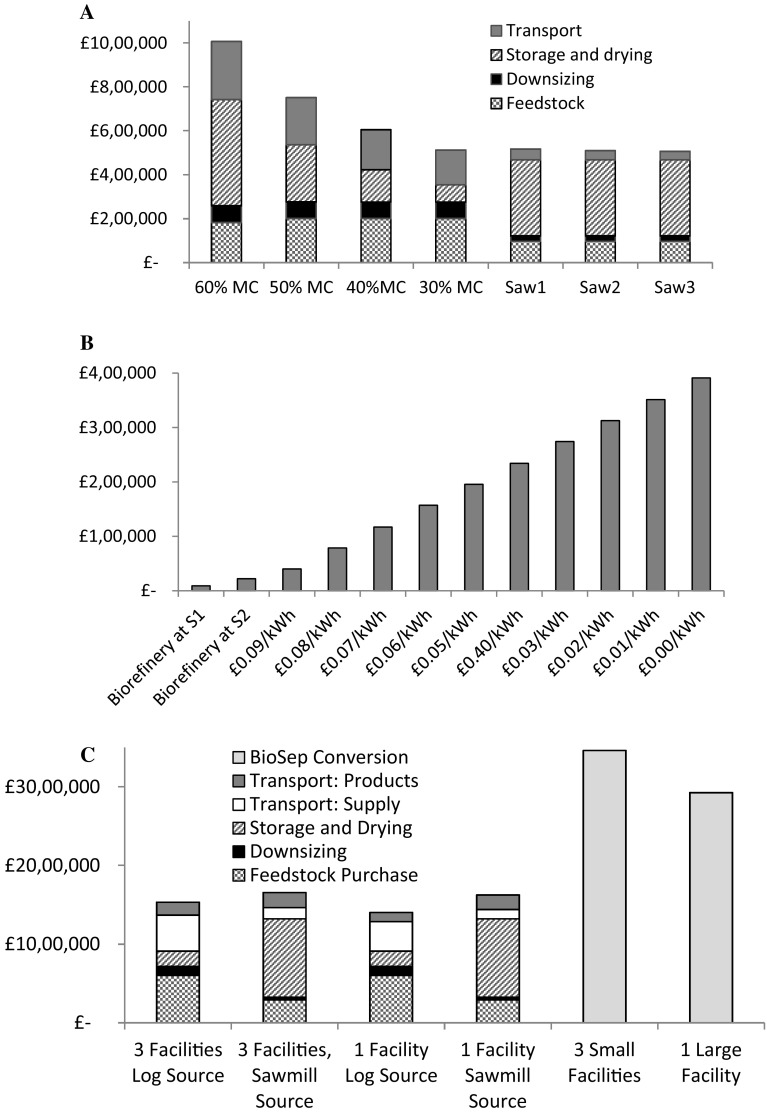
Analysis of supply chain optimisation results. **a** Cost contributions using minimum supply with different moisture contents (from L5) compared to the three sawmill chip sources. **b** Amount saved compared to the base case of the biorefinery located at the power station (heat cost £0.10/kWh) compared to located at S1 and S2 (heat cost £0.10 kWh) Then compared to savings when decreasing the cost of the heat source in the biorefinery. **c** Comparison of relative cost contributions in various scenarios using centralised versus distributed facilities. For clarity the cost of the processing configurations are shown separately

The main cost to the supply chain was the biorefining, (>50%) followed by drying (up to 25%) and feedstock transport (5–20%). Overall, sawmill by-products (55% MC) were cheaper than green saw logs (60% MC) and the southern sawmill (W3) was the preferred option for a feedstock source due to proximity to the processing sites (Fig. 2, sawmill chip supply chain). However, passively dried sawn logs were competitive once they reached 30% MC due to transportation and drying cost reduction. A comparative breakdown of the relevant costs are shown in Fig. 6a. Pre-processing, such as chipping, drying and milling was done at either the log storage or collection location to minimise transportation costs due to the reduced weight of the dried chips or logs. This finding is in agreement with past work on lignocellulosic biorefinery supply chains which demonstrated that downsizing and reduced moisture content, prior to transport was beneficial due to reduced transportation costs from higher density feedstock [4]. Due to the higher transportation costs and requirements in log processing, here the change in moisture content is a more significant factor for the log source compared to the sawmill source.

Then, analysis was completed in consideration of a fixed source using the supply driven model. The model was constrained to use a minimum of the equivalent to 1000 dry tons of chips from each sawmill (55% MC), and compared to using the equivalent to 1000 dry tonnes from log source five (L5) with various moisture contents with the availability of one biorefinery, using heat at £0.05/kWh. The comparative cost contributions to the supply chain are shown in Fig. 6a. The difference of using the optimal sawmill, 3, (M3) compared to sawmill 1 (M1) or 2 (M2) is minimal when there is only one biorefinery allowed as the capacity of the biorefinery dictates the economies of the supply chain rather than the capacity of the supply. Similarly, when sawmill one or two is used, all pre-processing is performed at the sawmill due to reduced transportation costs. Then for sawmill 1, biorefinery location 2 (B2) is used and products are sold in Aberdeen (C2). For sawmill two, biorefinery location 3 (B3) is used, and the products are sold in Glasgow (C1).

The moisture content reduction of forest logs (60–30% MC) had a significant effect on the supply chain in this case. Similar results are seen when considering sawmill by-products with lower moisture contents, however in the case of passive drying chips, significant losses in dry matter would be expected, unlike sawnlogs [27]. Previous analysis of moisture content effects has demonstrated the importance of this consideration for other lignocellulosic feedstocks (Miscanthus biomass), although ambient drying without loss of dry matter could not be facilitated [7]. In the case of Miscanthus, the material was utilised for heating and thus the moisture content effected the heating value of the material, as well as the transportation costs. Ambient drying of sawnlogs is expected to further reduce the cost of the downsizing of biomass with chippers being able to perform optimally with lower moisture contents [28]. Hence, as demonstrated here, moisture content can affect many aspects of the supply chain including pre-processing costs, transportation and configuration.

#### Biorefinery Location and Excess Heat from the Power Plant versus Electrical Heat

The biorefineries were placed next to potential sources of excess heat and hence the effect of heating cost on the overall supply chain was evaluated. This was designed to simulate different energy sources with variations in energy prices, available at different locations. When going from £0.10–£0.0 to £0.01/kWh the contribution of the cost of the biorefining process decreased from 74 to 72–69% when considering the optimal sawmill chip source as the feedstock. In the case of the sawmill source, the transport cost contribution is low compared to other costs, however the transportation cost is more significant in the case of the sawnlogs. Therefore, to further investigate the potential effect of the integration of the supply chain with the techno-economic model, the options were changed so that the biorefinery could either be co-located at the power station (B3 in Fig. 2) with a variable heat cost between £0.01 to £0.10/kWh or at the same site as either of the log storages (S1 or S2) with a heat cost of £0.10/kWh. In both cases, for location at S1 or S2, compared to the base case of the nearest power station, the savings with reduced heat energy cost are higher than the savings from reduced transportation costs. When storages were used as a biorefinery location, chipping, drying and milling were all located at the same site as the storage. Then for storage, S1, the log source used was L5, and for storage, S2, L4 was used then L2 due to the proximity to the storage location.

The approach of considering resources and technology implications simultaneously can be useful for planning and improvements of technology development. Previously, an integrated approach was used for first generation ethanol from sugar cane, and the approach enabled decisions about compromising situations between sugar and ethanol processes [37]. Similarly in the work presented here, technology configurations were considered with respect to the supply chain. The knowledge developed about the impact of the choice of heat energy source can subsequently inform decisions regarding heat integration and processing requirements such as temperatures in the development of the biorefining technology.

#### Demand Driven Model—Centralised versus Dispersed

The biorefinery facility considered so far has been a smaller scale facility, however it is of interest to compare to a theoretical larger facility with respect to demand. Therefore, centralised, larger facility versus several smaller facilities based on the case study at hand was investigated using the demand driven model. In this case a crude estimate of the capital costs was made using the point six power rule in a similar manner to previous distributed versus centralised optimisation [2]. A facility three times the size of the original facility was compared to three smaller facilities. To investigate this, a demand of 900 tonnes of cellulose at Glasgow and Aberdeen was set to be provided by either set of facilities. The base case scenario used was with 30% MC logs available because at higher moisture contents, the sawmill source was preferred. The relative results of analysis and contributing costs are displayed in Fig. 6 and Table 6 shows the configuration of the supply chain for logs and chips. As can be seen, in this situation the centralised, singular facility is preferable, both in consideration of the supply chain as a whole and in consideration of processing costs. Previous work has resulted in the preference of distributed pre-processing [2] and centralised bio-chemical plants, [8] although either choice is dependent on distances, transport and processing costs. In this work, the technology is the dominant cost contributing toward the supply chain which outweighs the benefits of distributed processing facilities and the subsequent reduction in transportation.

**Table 6:** Comparison of three small facilities to one large facility capable of processing an equivalent amount of biomass

	Three small conversion facilities		One large conversion facility	
	Log source	Sawmill source	Log source	Sawmill source
Collection points	West forest (L5) to south storage (S1)	Sawmill 3 (M3) provides all biomass	West forest (L5) to south storage (S1)	Sawmill 3 (M3) provides all biomass
Pre-processing	Located at storage (S1)	Located at sawmill (M3)	Located at storage (S1)	Located at sawmill (M3)
Biomass conversion and delivery	Glasgow receives from B1 and B3 Aberdeen receives from B2, plus some cellulose from B1	Glasgow receives from B1 and B3 Aberdeen receives from B2, plus some cellulose from B1	Conversion facility located at B3	Conversion facility located at B1
Profit	£2,695,211	£2,598,080	£3,280,121	£3,139,455

### Implications

The results of this work have demonstrated the importance of moisture content, pre-processing and heat energy consideration with respect to the whole supply chain. Future configurations of the supply chain should incorporate moisture content and downsizing effects into the model in a similar manner as moisture content, bulk density and heating value has previously been incorporated [3]. Here, as we were using Excel Solver Foundation, this was able to be calculated for our case study but it would be beneficial to automate this throughout the model. In addition, since drying costs were so significant, future work should focus on passive drying for woodchips, the incorporation for waste heat as a means of drying and technology choices using wet biomass. These options will have subsequent implications on the supply chain which must also be evaluated. If considering drying chips the model must also incorporate dry matter loss in regards to the total biomass conversion [6].

The availability of various product streams provides a generic framework, useful for the evaluation of a variety of lignocellulosic products in the multi-product biorefinery setting. The model can be useful for comparing and identifying the key bottlenecks to consider with respect to profitability with regards to decision making for a biorefinery with multiple feedstock and product options. In addition the incorporation of the technology choices within the supply chain decision making, allows key bottlenecks for the technology to be considered on a holistic scale. However, once all of this is implemented the environmental impact must also be evaluated.

## Conclusion

The flexibility of the optimisation model allowed for analysis of key parameters such as moisture content, feedstock choices, downsizing and drying options, and location effects for the technology configuration. The incorporation of the technology assessment with the optimisation model enabled a holistic evaluation of the conversion technology in an identified setting. Once the key bottlenecks were identified within the technology, ways to overcome these costs could be further evaluated with respect to the supply chain. The optimisation model provides a generic framework for scenarios where one technology may consider more than one simultaneous feedstock type, pre-processing route and processing configuration. The significant variation in costs given the alternative scenarios demonstrated the importance of this flexibility in consideration of biorefinery decision making. The integrated cost analyses are then able to assist in the focussing of technology development and investment choices for the biorefining industry.

## Appendix

See Table 7.

**Table 7:** Processing costs and energy requirements

Item	Information and energy requirements	Estimated capital cost
Slurry preparation		
Sawdust feed	1 kW/t	=16.9WL = $64 896
Acidic water mix and feed	0.5 kW/t	USD 10,500
Circulation and feed forward pump	40 kg/min (2.4 t/h) pre-treated slurry 4 kW	£10,000
Vacuum pump	1–2 kW	£1000
Heat input	Calculated energy. Steam system	USD 100,000
Stirrer/agitation	1–2 kW	$40,000 (including stirrer)
Blender	4 kW	$40,000 (including stirrer)
De-aeration	30 min, 1200 kg 6 kW	£18,000
Organosolv mix and feed	0.5 kW/t	USD 10,500
Sonication		
Circulation pump	7 kW (max) 80 kg/min (4.8 t/h)	£10,000
Heat input	Calculated	
Stirrer	1–2 kW	USD 10,500
Ultrasound	2500 kg mixture, power for 10 min is 100–300 kW i.e. 800 kG in 10 min is 100 kW - based on upper limit	€2.5 M + €450 K/2–3 years
Centrifuge	80 kg/min 6.5 kW	$USD 60,000
Organoslv addition	0.5 kW/t	USD 10,500
Heating and cooling	Calculated	USD 100,000
Separation		
Solid drying	Calculated based on heat requirement to change temperature to 80 °C	USD 5000
Water addition to liquid	0.5 kW/ton	USD 10,500
Organic/aqueous separation	1 kW/ton	USD 20,000
Lignin extraction	1 kW/ton	USD 100,000
MIBK recovery	1000 kJ/kg = 1 kW/t	USD 50,000
Sugar extraction	10 kW/ton	USD 100,000
EtOH recovery	840 kJ/kg = 0.84 kW/t (8 Nov from Bio-Sep)	USD 50,000
Acidic water recovery	2260 kJ/kg = 2.26 kW/t (8 Nov from Bio-Sep)	USD 50,000
Circulation pump	This was based on previous information fed about circulation pumps and included in all processes except for solid drying	£10,000

See Table 8.

**Table 8:** Material costs and values

Material	Price	Source
MIBK	USD 1800/ton (£1116)	US$1600–$1800/ton http://uk.alibaba.com (Accessed 26-10-13)
Ethanol	£500/ton	US$800–900/ton, US$500 http://www.alibaba.com/ (Accessed 28-10-13)
Organic acid	£700/ton	US$650–700/ton (Accessed 28-10-13) http://uk.alibaba.com/product/120981138-Oxalic-acid.html
Water	£0.5629 per ton plus £121 fixed price per year	http://anglianwater.co.uk/business/your-account/tariffs/streamline-blue/index.aspx £600 fixed plus £1.0420/ton. Non potable water. (Accessed 26-10-13)
Waste disposal		
Organic acid neutralisation with HNaCO_3_	Cost of HNaCO_3_ is about USD 230 = £140	Ratio of baking soda to oxalic acid for neutralisation is 1.87 (mole ratios) so this is included in the model and used to neutralise the remaining organic acid Organic acid + 2.baking soda = salt + 2.water + 2.carbon dioxide

### Other Fixed and Operational Costs

Labour has been estimated based on a £6 minimum wage to be £10 and £20 per hour for the labourers and supervisor respectively, (variable). Electricity and heating costs were estimated based on domestic prices found online to be £0.1 and £0.05 per kWh, respectively. Maintenance costs, unless otherwise specified are based on 2% of capital costs as per methods employed by the National Renewable Energy Laboratory (NREL).
